# QTL mapping for leaf rust resistance in bread wheat using PBW343/W8627 RIL population

**DOI:** 10.3389/fpls.2026.1851529

**Published:** 2026-07-01

**Authors:** K. S. Vindhya, Renu Sharma, Satish Kumar, Pramod Prasad, Prem Lal Kashyap, Rajender Singh, M. Sivasamy, Sripada M. Udupa, Indu Sharma, Ratan Tiwari, Pradeep Sharma

**Affiliations:** 1 Indian Council of Agricultural Research (ICAR)–Indian Institute of Wheat and Barley Research, Karnal, India; 2 Indian Council of Agricultural Research (ICAR)- Indian Institute of Wheat and Barley Research Regional Station, Shimla, India; 3 Indian Council of Agricultural Research (ICAR)- Indian Agricultural Research Institute Regional Station, Wellington, Tamil Nadu, India; 4 International Center for Agricultural Research in the Dry Areas (ICARDA), Rabat, Morocco; 5 Department of Plant Pathology, Punjab Agricultural University (PAU), Ludhiana, India

**Keywords:** all stage resistance (ASR), bread wheat, DArT, leaf rust, QTLs

## Abstract

**Introduction:**

Leaf rust, caused by *Puccinia triticina*, inflicts 10-50% yield losses in bread wheat, with newly emerging virulent pathotypes overcoming major *Lr* genes and rendering the cultivars susceptible. The identification of genetic loci conferring resistance to wheat leaf rust, coupled with the development of molecular markers and the incorporation of resistant alleles into breeding programs, represents the most sustainable and cost-effective strategy for disease management.

**Methods:**

The present study, employed a recombinant inbred line population derived from (PBW343/W8627) was utilized to map QTLs for leaf rust resistance at seedling and adult plant stages. The RIL population along with its parents was evaluated at seedling and adult plant stages against most prevalent and virulent pathotypes at seedling and adult plant stages for the two crop seasons (2021-22 and 2022-23).

**Result:**

Analysis of variance revealed significant differences among the RILs across years. For QTL mapping, genotyping was conducted using 3.9K DArT SNPs leading to the identification of two QTLs: *QLr.iiwbr.2B.1* (2B) explaining phenotypic variance of 13.26% with a LOD score 8 and *QLr.iiwbr.5B.1* (5B) explaining 14.91% with LOD score 3.91 for all stage resistance (ASR) as well as one QTL for adult plant resistance (APR), *QLr.iiwbr.6B.1* (PVE 14.21%). Further, *in silico* analysis revealed a putative candidate gene encoding for Annexin (*TraesCS5B02G201700*) which plays an important role in disease resistance in wheat.

**Discussion:**

The study identified new QTLs associated with leaf rust resistance, providing valuable sources to accelerate the development of durable rust resistant sources. Further, characterization of these QTLs will strengthen their utility in rust resistance breeding.

## 1 Introduction

Bread wheat (*Triticum aestivum* L.) ranks among the world’s most important cereal crops grown worldwide. Ensuring stable and sustainable wheat production is therefore necessary for global food security amid rising population, climate variability, and biotic stresses. Among the major diseases affecting wheat production, rust diseases pose a persistent and serious threat, with leaf rust being the most widespread and frequently occurring (Kang et al., 2024). Caused by the obligate biotrophic fungus *Puccinia triticina* Eriks., leaf rust affects almost all regions where wheat is cultivated and can result in significant yield and quality losses. Under favorable environmental conditions, yield reductions of 10-20% are common in susceptible cultivars, while losses may exceed 50% during severe epidemics (Savary et al., 2019). In addition to yield reduction, leaf rust adversely affects grain quality by reducing kernel weight and test weight (Draz et al., 2015). The pathogen exhibits a high degree of adaptability and genetic variability, leading to the frequent emergence of new virulent pathotypes that overcome deployed resistance genes (Wang et al., 2025).

In India, leaf rust is endemic nationwide across diverse agro-climatic regions including the Northern Plains Zone (Punjab, Haryana, Uttar Pradesh, and Bihar), central India (Madhya Pradesh and Maharashtra), with relatively higher disease pressure and peninsular states (Karnataka, Telangana, Andhra Pradesh, and Tamil Nadu) posing a persistent threat to wheat productivity (Gautam et al., 2021). Extensive national rust surveillance has revealed considerable pathotype diversity in *Puccinia triticina* populations in India. During 2008-2013, a total of 37 pathotypes were identified, among which 121R63-1 (77-5), 121R60-1 (77-9), 21R55 (104-2), and 21R63 (104-3) were the most prevalent and widely distributed. Earlier studies reported 121R63-1 (77-5) as the dominant pathotype in central and peninsular India; however, subsequent surveillance indicated that 121R60-1 (77-9) has emerged as the most widely distributed pathotype across major wheat-growing regions of the country. Many of these predominant pathotypes exhibit virulence against commonly deployed leaf rust resistance (*Lr*) genes, underscoring the dynamic nature of the pathogen population and the vulnerability of existing resistance sources (Bhardwaj et al., 2016; Lohate et al., 2008; Gupta et al., 2017).

Host plant resistance is widely recognized as the most economical, environmentally safe, and sustainable strategy for managing leaf rust. To date, more than 80 leaf rust resistance (*Lr*) genes have been identified and catalogued in wheat and its wild relatives (Singh et al., 2024). However, a large proportion of these genes confers race-specific, seedling-stage resistance and often loses effectiveness within a short period due to rapid pathogen evolution (Ren et al., 2023). The breakdown of resistance in widely grown cultivars such as PBW 343 and the erosion of resistance conferred by previously durable genes like *Lr19* highlight the limitations of relying on single major resistance genes (Sharma et al., 2021). In contrast, adult plant resistance (APR), typically governed by multiple minor genes, is generally race non-specific and more durable across environments. APR reduces pathogen multiplication and slows disease development, thereby exerting lower selection pressure on the pathogen population. Consequently, APR has gained increasing importance in wheat breeding programs aimed at achieving durable resistance (Figlan et al., 2024).

Quantitative trait loci (QTL) mapping has emerged as a powerful approach to dissect the genetic basis of complex traits such as leaf rust resistance. QTLs often represent genomic regions controlling partial or slow-rusting resistance and are less prone to breakdown compared to major resistance genes. The identification of stable QTLs across environments and genetic backgrounds is therefore crucial for the development of wheat varieties with durable leaf rust resistance. Recent advances in molecular marker technologies and high-density genetic maps have significantly improved the precision of QTL detection in wheat. Marker systems such as SSRs, SNPs, and Diversity Arrays Technology (DArT), along with high-throughput genotyping platforms, have enabled dense genome coverage suitable for the complex hexaploid wheat genome (Schouten et al., 2012). Marker-assisted selection (MAS) and genomic tools facilitate the pyramiding of multiple resistance QTLs, thereby enhancing the durability and effectiveness of resistance breeding programs. Moreover, the identification of resistance-associated genomic regions aids in the discovery of candidate genes and accelerates wheat improvement efforts (Kilian et al., 2005; Manickavelu et al., 2011). Additionally, over 200 QTLs have been discovered and summarized for leaf rust resistance across 21 wheat chromosomes till date (Zhang et al., 2025).

In this context, the present study entitled “QTL Mapping for leaf rust resistance in bread wheat” was undertaken to identify QTL(s) associated with leaf rust resistance evaluated under field and controlled conditions. The identification of reliable and stable QTL(s) linked to leaf rust resistance will contribute to a better understanding of the genetic architecture of resistance and provide valuable molecular resources for the development of durable leaf rust-resistant wheat varieties.

## 2 Materials and methods

### 2.1 Plant material

The plant material comprised a RIL population (F_8_) of 212 lines derived from the cross between W8627, a confirmed multiple disease resistant genetic stock (Virdi et al., 2016) and susceptible, agronomically superior mega-variety PBW343 was evaluated for leaf rust resistance.

### 2.2 Greenhouse seedling evaluation

Seedling resistance of the parental lines and the RIL population along with both the parents and differential sets were raised in a sterilized growing medium consisting of fine loam soil and well-decomposed farmyard manure (FYM) mixed in a 3:1 ratio. The soil mixture was autoclaved at 121 °C and 15 psi for 60 min, and sowing trays were disinfected with 2% Lysol solution prior to use. Aluminum trays (29 × 12 × 7 cm) were used to sow seeds at a depth of approximately 1 cm. Seedlings were maintained in spore-proof chambers at 20 ± 2 °C under adequate light conditions. Initial seedling screening was performed on parental lines W8627 (resistant) and PBW343 (susceptible) using a panel of seven *P. triticina* pathotypes: 12-5 (29R45), 12-7 (93R45), 77-1 (109R31-1), 77-5 (121R63-1), 77-9 (121R60-1), 104-1 (21R31-1), and 104-2 (21R55). Seedlings were inoculated following the standard rust inoculation protocol. Based on contrasting infection responses between the parents, three pathotypes (77-5, 77-1, and 12-7) were selected for further screening of the 212 RILs. The virulence/avirulence details for these three pathotypes are given in **Table 1**.

**Table 1:** Virulence/Avirulence formulae for the distinct pathotypes.

S. No.	Pathotypes Name		Avirulence	Virulence
	Old	New		
1	77-1	109R63	*Lr9*, *Lr17*, *Lr17a*, *Lr17b*, *Lr19*, *Lr23*, *Lr24*, *Lr25*, *Lr27, Lr 31*, *Lr28*, *Lr29*, *Lr32*, *Lr36*, *Lr39*, *Lr42*, *Lr43*, *Lr45*, *Lr47*, *Lr53*, *Lr57*, *Lr58*, *Lr61*, *Lr62*, *Lr72*, *Lr80*	*Lr1*, *Lr2a*, *Lr2b*, *Lr2c*, *Lr3*, *Lr10*, *Lr11*, *Lr12*, *Lr13*, *Lr14a*, *Lr14b*, *Lr14ab*, *Lr15*, *Lr16*, *Lr18*, *Lr20*, *Lr21*, *Lr22a*, *Lr22b*, *Lr26*, *Lr30*, *Lr33*, *Lr34*, *Lr35*, *Lr37*, *Lr38*, *Lr44*, *Lr46*, *Lr48*, *Lr49*, *Lr51*, *Lr52*, *Lr65*, *Lr67*
2	77-5	121R63-1	*Lr9*, *Lr18, Lr19*, *Lr24*, *Lr25*, *Lr28*, *Lr29*, *Lr32*, *Lr39*, *Lr42*, *Lr43*, *Lr45*, *Lr47*, *Lr53*, *Lr57*, *Lr61*, *Lr62*, *Lr80*	*Lr1*, *Lr2a*, *Lr2b*, *Lr2c*, *Lr3*, *Lr10*, *Lr11*, *Lr12*, *Lr13*, *Lr14a*, *Lr14b*, *Lr14ab*, *Lr15*, *Lr16*, *Lr17a*, *Lr17b*, *Lr20*, *Lr21*, *Lr22a*, *Lr22b*, *Lr23*, *Lr26*, *Lr27*, *Lr30*, *Lr33*, *Lr34*, *Lr35*, *Lr36*, *Lr37*, *Lr38*, *Lr40*, *Lr44*, *Lr48*, *Lr49*, *Lr51*, *Lr52*, *Lr58*, *Lr65*, *Lr67*, *Lr72*
3	12-7	93R45	*Lr1, Lr2a, Lr9, Lr13, Lr15, Lr19, Lr24, Lr25, Lr28, Lr29, Lr32, Lr36, Lr39, Lr42, Lr43, Lr45, Lr47*	*Lr2b, Lr2c, Lr3, Lr10, Lr11, Lr12, Lr14a, Lr14b, Lr14ab, Lr16, Lr17a, Lr17b, Lr18, Lr20, Lr21, Lr22a, Lr22b, Lr23, Lr26, Lr27, Lr31, Lr30, Lr33, Lr34, Lr35, Lr37, Lr38, Lr40, Lr44, Lr46, Lr48, Lr49*

For RIL evaluation, seedlings were raised in metal trays accommodating 10 rows per tray, including one row of the susceptible check variety ‘Agra Local’. Seven-day-old seedlings were inoculated with uredospores suspended in Soltrol 170 using an atomizer to ensure uniform spore deposition. Following inoculation, trays were incubated in a humid chamber at 20 ± 2 °C with a 16 h light/8 h dark photoperiod for 24 h to facilitate infection and subsequently transferred to a greenhouse maintained at 15 ± 1 °C for disease development. Elemental sulphur dust was applied after inoculation to prevent powdery mildew contamination (Prasad et al., 2021). Inoculations were considered successful when the susceptible check consistently exhibited infection types (ITs) of 3+ to 4. Seedling infection types were recorded 14 days after inoculation using the 0-4 scale described by Nayar et al., 1997) wherein (i) 0;,;/;-,;1, 2- = Resistant; (ii) 12, 12+, 2+ = Moderately Resistant; (iii) 3, 23 = Moderately susceptible, (iv) 3+, 33+ = Susceptible for QTL analysis.

### 2.3 Field evaluation for adult plant resistance

The RIL population along with parental lines W8627 and PBW343 was evaluated for adult plant resistance (APR) to leaf rust for the two consecutive crop seasons 2021-22 and 2022-23 at ICAR-IIWBR, New Seed Farm, Karnal, India. Sowing was carried out under irrigated conditions. Standard agronomic practices recommended for the region were followed. The susceptible wheat variety Agra Local was sown after every 20 rows of the test material for consistent infection of disease. Border infector rows comprised a mixture of susceptible varieties: WL711, Agra Local, Kharchia, and C306 were planted across the periphery of the field. Leaf rust epiphytotic conditions were created using a mixture of virulent *P. triticina* pathotypes. Fresh uredospore inoculum was obtained from ICAR-IIWBR Regional Station, Flowerdale, Shimla, and multiplied at ICAR-IIWBR, Karnal. The inoculum was prepared by suspending uredospores in water containing a few drops of Tween-20 to facilitate uniform dispersion (Prasad et al., 2021)). Artificial inoculation was carried out using an ultra-low volume (ULV) sprayer during evening hours on alternate days until visible symptoms appeared on the susceptible check.

Disease progression was monitored weekly from February to late March. Leaf rust severity was recorded using the modified Cobb’s scale (Peterson et al., 1948). Host responses were classified as Immune, I (0.0), resistant, R(0.2), moderately resistant, MR, (0.4, intermediate, X (0.6), moderately susceptible, MS (0.8), and susceptible, S (1.0) based on uredia size and associated necrosis or chlorosis, following standard rust scoring guidelines (Roelfs et al., 1992). Successful inoculation was confirmed by consistent development of severe rust symptoms on the susceptible check ‘Agra Local’, which served as a reference. The coefficient of infection (CI) and area under disease progression curve (AUDPC) was calculated as described in the prior publication (Sharma et al., 2026).

### 2.4 Phenotypic data analysis

Analysis of variance was performed in R version 4.3.2 (R Core Team, 2015) using the ‘Agricolae’ package to partition genetic and environmental effects among the RILs. Raincloud and violin plots were created with ‘ggplot’ package and the mean, the mean of Coefficient of Infection (CI) of both years was used to assess the genetic effects and identify the QTL.

### 2.5 Genotyping, and SNP filtering

**Figure 1:**
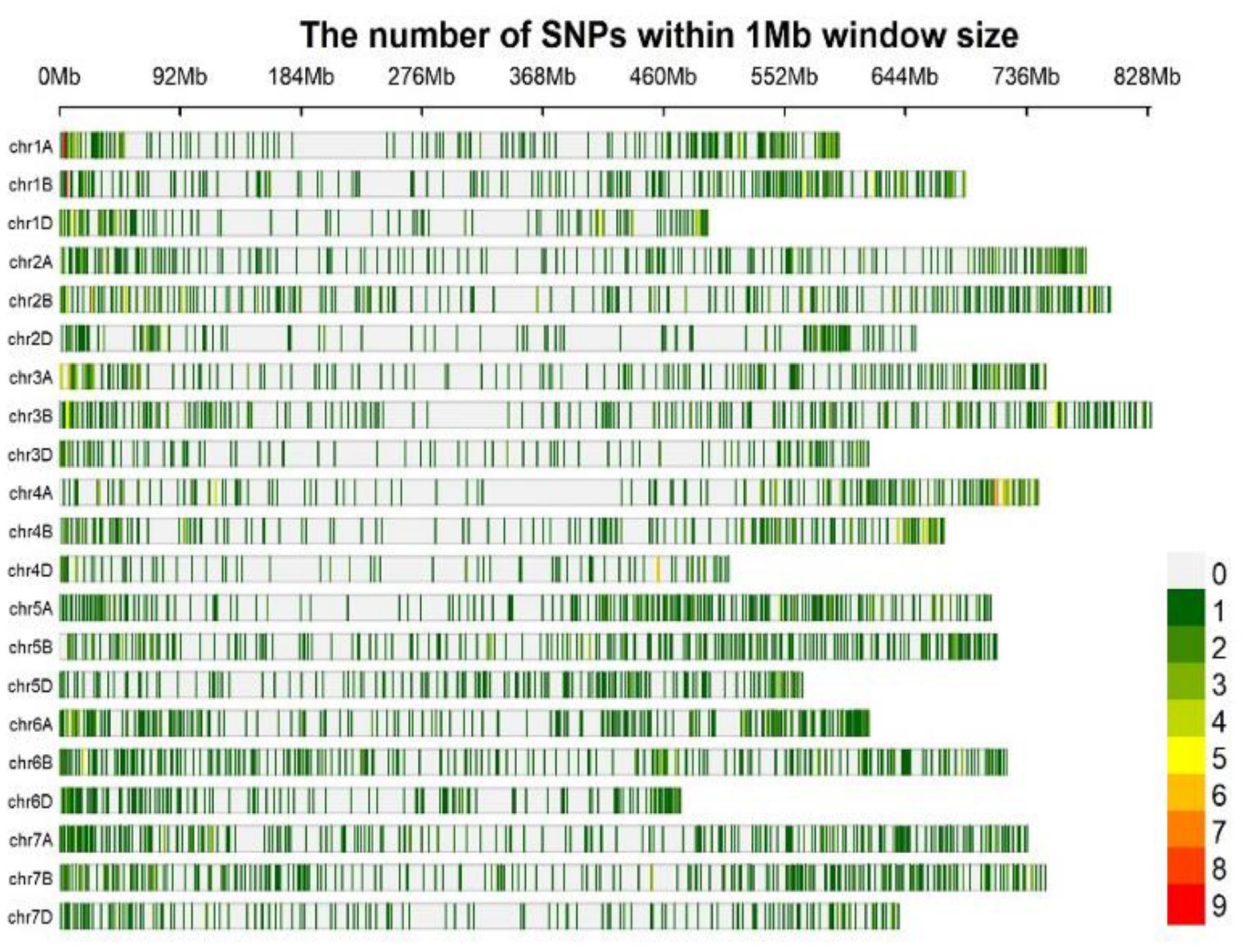
SNP density plot for 3.9K SNPs in the Wheat DArT AG panel. (Source: Wheat 3.9K mid-density genotyping services | Excellenceinbreeding).

Genotyping was performed using a comprehensive marker panel of 3, 897 DArT SNP markers distributed across all 21 wheat chromosomes (**Figure 1**). Markers were primarily derived from CIMMYT elite lines and the 90K iSelect SNP array (Velu et al., 2016; Sehgal et al., 2020). Raw data was subjected to stringent filtering using the dartR package (Jombart and Caitlin, 2015; Gruber et al., 2018) as described in our previous publication (Sharma et al., 2026). The physical positions of the DArT SNP markers were determined with reference to the RefSeq v1.0 sequences (https://excellenceinbreeding.org/toolbox/services/wheat-3.9k-mid-density-genotyping services-0). The resulting high-quality marker set was used for linkage mapping and QTL analysis.

### 2.6 Linkage map construction and QTL analysis

From the initial 3, 897 DArT markers, 602 high-quality markers meeting filtering criteria were used to construct the genetic linkage map of the RIL population. Linkage mapping and QTL detection were performed using IciMapping v4.2 (Wang et al., 2019), employing the Kosambi mapping function (Kosambi, 2017) to convert recombination frequencies to genetic distances. Marker ordering utilized the 2-opt optimization algorithm with rippling at a 5 cM window. QTL mapping was conducted using the inclusive composite interval mapping additive model (ICIM-ADD) (Li et al., 2008) with a LOD threshold of 3.0. Genome-wide scans used a walking speed of 1.0 cM, and significance thresholds were determined through 1, 000 permutations maintaining a genome-wide error rate of 0.10 and type I error rate of 0.01. Identified QTLs were named following the convention: “QTL + trait + research department + chromosome”.

### 2.7 *In silico* analysis

Putative candidate genes and their molecular functions were identified by analyzing flanking marker sequences associated with stable QTLs, using the RefSeq v1.0 wheat genome assembly from the International Wheat Genome Sequencing Consortium (IWGSC, 2018). Sequence homology searches were conducted with the BLASTN algorithm integrated into the Ensembl Plants database (https://plants.ensembl.org/index.html) Functional annotation of the candidate genes was subsequently validated through comprehensive review of published literature.

## 3 Results

### 3.1 Seedling resistance test

Parental screening was initially conducted using seven distinct pathotypes of *P. triticina* to assess the differential seedling-stage responses of the two parental lines: W8627 (resistant) and PBW343 (susceptible). The pathotypes included:77-9, 77-5, 104-2, 77-1, 12-7, 12-5, and 104-1. The screening revealed that both parents were susceptible to the majority of the pathotypes. However, notable differences were observed in their specific reaction profiles (**Table 2**).

**Table 2:** Percentage distribution of RILs showing different infection responses to *P. triticina* pathotypes.

Pathotype	Genotypes		Total number of RILs	%R	%MR	%MS	%S
	W8627	PBW343					
77-5	R	S	212	8.96	0.94	3.30	86.79
77-1	R	S	212	21.23	1.42	6.60	70.75
12-7	R	S	212	39.62	7.08	4.72	48.58

The Donor parent W8627 was susceptible to 77-9 and 104-2, but displayed clear resistance to 77-5, 77-1, 12-7, 12-5, and 104-1. Conversely, the recipient parent PBW343 was susceptible to 77-9, 104-2, 77-1, and 12-7. These findings suggest that W8627 harbors unique resistance factors, especially effective against 77-5, 77-1, and 12-7, which are absent in PBW343. The contrasting reactions, particularly to pathotype 77-5, formed the basis for selecting this pathotype along with 77-1 and 12-7 for further evaluation in the RIL population. This selection aimed to facilitate the identification of genomic regions associated with seedling stage leaf rust resistance. Based on their ability to distinctly differentiate the parental lines, pathotypes 12-7, 77-1, and 77-5 were selected for screening the 212 RILs developed from the cross W8627 × PBW343.

**Figure 2:**
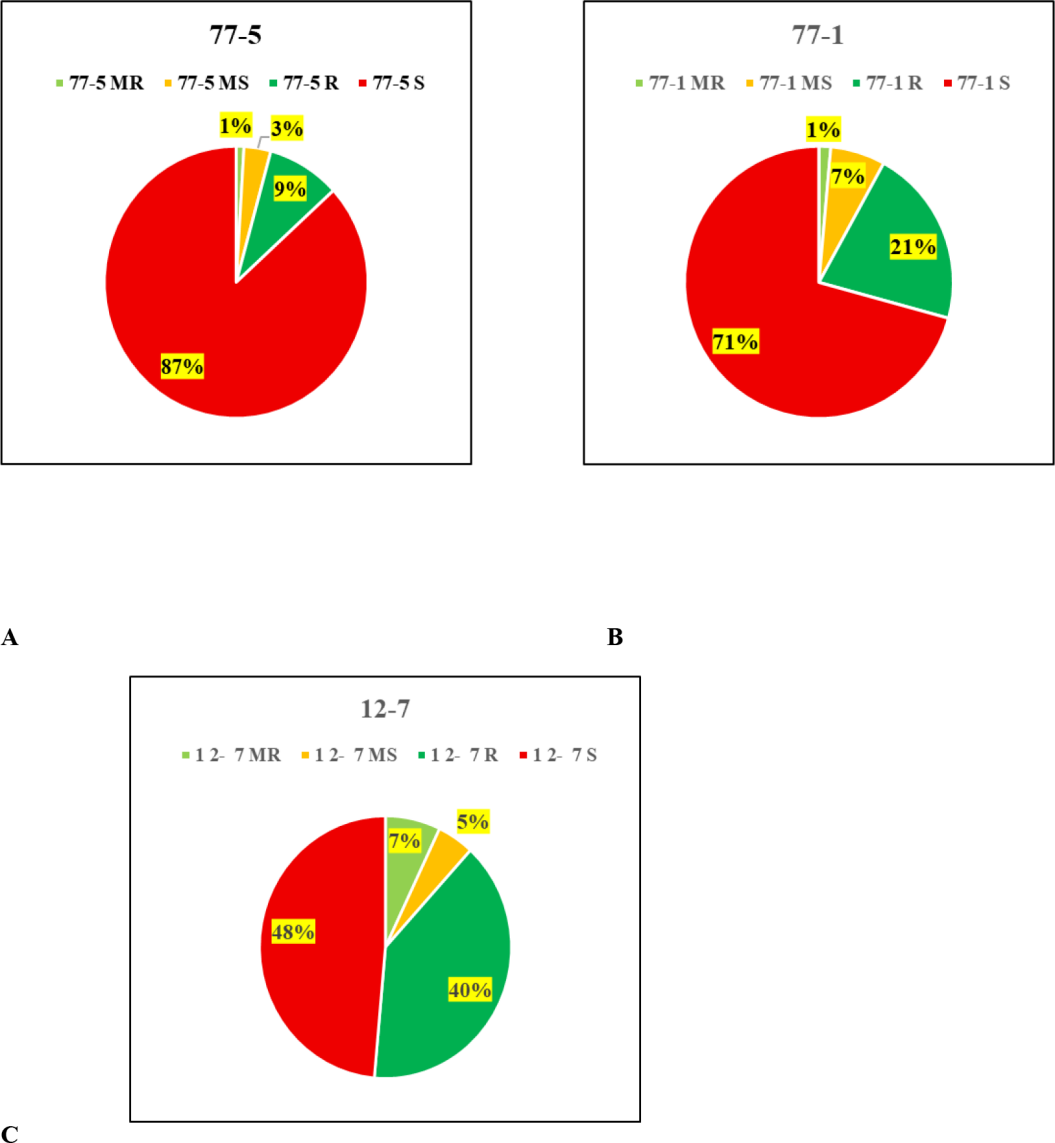
Infection type among 212 RILs at the seedling stage upon infection with 3 distinct pathotypes: **(A)**: 77-5, **(B)**: 77-1, **(C)**: 12-7; R, Resistant; MR, Moderately resistant; MS, Moderately Susceptible; S, Susceptible.

**Figure 3:**
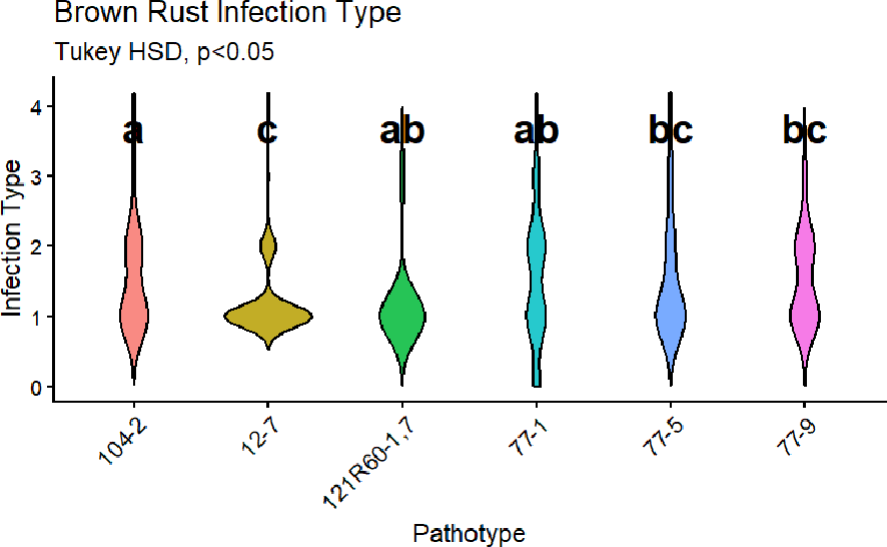
Violin plots showing leaf rust infection type (0-4 scale) across six pathotypes in RIL population. Tukey HSD groupings (p<0.05): pathotype 104-2(a, most resistant); 12-7 (c, most virulent); 121R60-1, 7, 77-1, 77-5, 77-9 (ab/bc, intermediate).

The results revealed significant variation in rust severity among the RILs, indicating clear segregation of resistance traits. The relative aggressiveness of the pathotypes, based on the extent of observed susceptibility, followed the order: 77-5 > 77-1 > 12-7 (**Figures 2**, **3**). Among these, pathotype 77-5 was the most virulent, causing high levels of disease in the majority of susceptible lines, whereas 12-7 exhibited comparatively lower virulence. The response of the RIL population to each pathotype was as follows: For pathotype 77-5, 8.96% of RILs were classified as resistant, 0.94% as moderately resistant (MR), 3.30% as moderately susceptible (MS), and 86.79% as susceptible. Against pathotype 77-1, 21.23% of RILs showed resistance, 1.42% were MR, 6.60% MS, and 70.75% susceptible. In response to pathotype 12-7, 39.62% of RILs were resistant, 7.08% MR, 4.72% MS, and 48.58% susceptible.

### 3.2 Field evaluation

**Figure 4:**
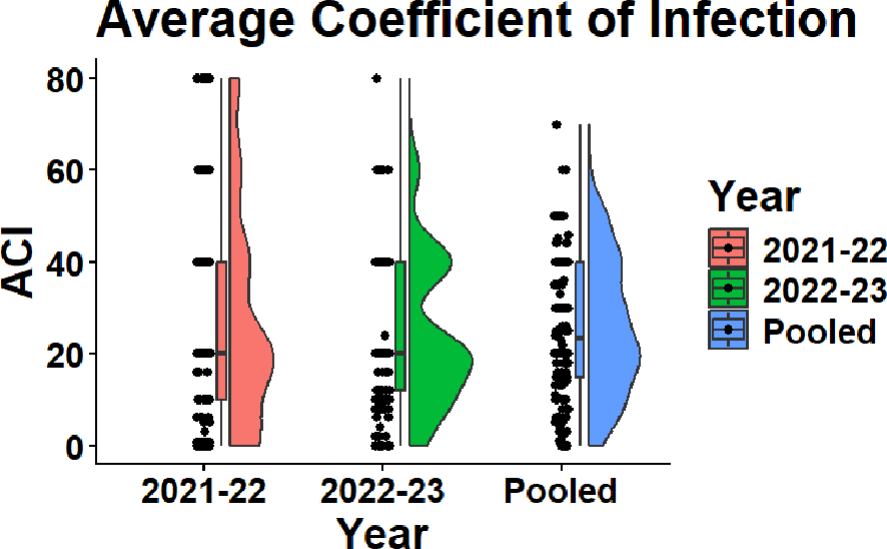
Raincloud plot depicting Average Coefficient of Infection (ACI) values for RILs and parents across 2021-22 and 2022-23 crop seasons, including pooled data.

Leaf rust response of both the parents and the RIL population were assessed at the adult plant stage during the 2021-22 and 2022-23 crop seasons. Across both years, W8627 consistently exhibited resistance, with ACI values of 2.9 and 2.6 and AUDPC values of 52 and 55. In contrast, PBW343 displayed moderate susceptibility, recording ACI values of 60 and 50 and AUDPC values of 460 and 580, respectively. Mean values for the resistant and susceptible parents (ACI = 2.75 and 53.5 and AUDPC = 55 and 520). Among the RILs, AUDPC values ranged widely from 0 to 860. Average AUDPC trends over time revealed a steady increase in both years, with a steeper trajectory in 2021-22, reflecting stronger disease pressure. The susceptible check, Agra Local, recorded an ACI of up to 80. A normal distribution of ACI values was observed among parents and RILs (**Figure 4**), confirming the suitability of the RIL population.

Furthermore, significant genotypic, environmental, and genotype-by-environment variances (*P* < 0.01) were analyzed, highlighting the wide variability among the RIL population for leaf rust resistance (**Table 3**).

**Table 3:** Variance components of disease severity scores of RIL population for the crop season (2021-22 and 2022-23).

Sources of variation	df	Mean square	F- value
RILs	211	966.068396	40.85169
Replicates/Environment	1	9.08681903	0.563868
Error	211	25.5828001	
***P<0.001			

### 3.3 Genotypic data filtering and construction of linkage map

After applying the standard data filtering protocols, a total of 1500 SNP markers were initially retained based on repeatability thresholds. Markers with low SNP call rates were subsequently removed, while none of the markers were excluded based on heterozygosity levels. Following this filtration, 1420 DArT markers were retained and further subjected to parental polymorphism analysis. Out of these, 602 DArT markers were identified as polymorphic between the two parents (W8627 and PBW343) (**Table S1**) and were also segregating in the RIL population. These 602 polymorphic markers were used for the construction of the genetic linkage map.

The resulting genetic map spanned a total length of 1395.52 centi Morgan (cM), with an average marker/bin interval of 0.46 cM. Among the chromosomes, 5D recorded the maximum length (144.23 cM), whereas 4D was the shortest (4.0 cM). Chromosome 2A, with 91 markers, covered a length of 125.43 cM, corresponding to a marker density of 0.73 markers per cM. Within the wheat genome, the A sub-genome contributed the highest proportion of markers (283 markers, 47%), while the D sub-genome contributed the least (83 markers, 13.78%). Analyzing the distribution across the seven homoeologous groups, the fifth group exhibited the highest number of SNPs (110, 18.27%), whereas the first group had the fewest (54, 8.97%). On average, each of the 21 wheat chromosomes harbored approximately 75 markers. Notably, chromosome 2A had the highest marker density (54 markers, 8.97%), while chromosomes 2D and 4D had the lowest number (7 markers each, 1.16%). The finalized genetic map comprised 21 linkage groups, each representing one of the 21 wheat chromosomes.

### 3.4 QTL analysis for seedling stage resistance

**Figure 5:**
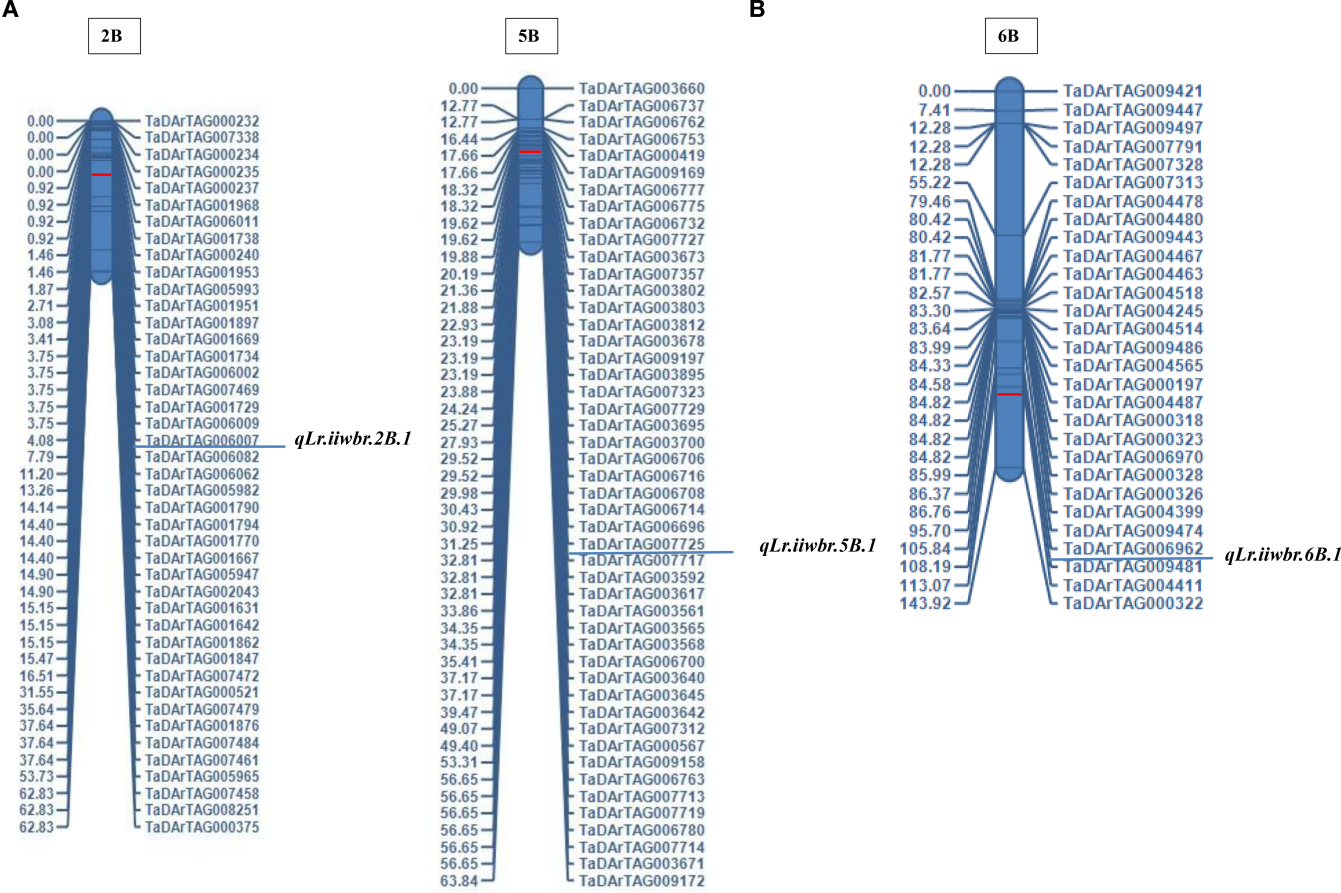
**(a)** Identified QTLs for all stage resistance (ASR) and their genomic locations: i) QTL on chromosome 2B i.e. *qLr.iiwbr.2B.1*; ii) QTL on chromosome 5B i.e. *qLr.iiwbr.5B.1.* **(b)** Identified QTL for adult plant resistance (APR) and its genomic location; i) QTL on chromosome 6B i.e. *qLr.iiwbr.6B.1*.

For all stage resistance, two important QTLs were identified. A QTL designated as *qLr.iiwbr.2B.1* was identified on chromosome 2B in response to pathotype 77-1. The locus exhibited a LOD score of 8.08 and accounted for 13.26% of the phenotypic variation (PVE). This QTL was located at 5.0 cM, flanked by the markers TaDArTAG006007 and TaDArTAG006082 (**Figure 5A**). A second QTL, *qLr.iiwbr.5B.1*, was detected in response to pathotype 77-5 on chromosome 5B, positioned at 32 cM, with a LOD score of 3.91 and PVE of 14.91%. The confidence interval for this QTL ranged from 31.5 cM (left CI) to 32.5 cM (right CI) and flanked by markers TaDArTAG007725 and TaDArTAG007717 (**Figure 5A**; **Table 4**).

**Table 4:** QTLs for leaf rust resistance showing chromosome, markers, position, LOD, CI, PVE%, and pathotype for seedling stage resistance.

QTL name	Chromosome	Left marker	Right marker	Position (cM)	LOD score	Left CI	Right CI	PVE %	Pathotype
*qLr.iiwbr.2B.1*	2B	TaDArTAG006007	TaDArTAG006002	5.00	8.08	4.08	7.79	13.26	77-1
*qLr.iiwbr.5B.1*	5B	TaDArTAG007725	TaDArTAG007717	32.0	3.90	31.5	32.5	14.91	77-5

### 3.5 QTL analysis for APR

In the case of adult plant resistance, an important QTL, i.e. *qLr.iiwbr.6B.1*, was identified on chromosome 6B using a linkage map constructed with 602 DArT markers. This QTL was flanked by two DArT SNP markers, TaDArTAG006962 and TaDArTAG0009481, and was located at 107 cM. It exhibited a LOD score of 3.8 and explained 14.219% of the phenotypic variance (**Figure 5B**; **Table 5**).

**Table 5:** QTL for leaf rust resistance showing chromosome, markers, position, LOD, CI, PVE%, and pathotype for adult plant resistance.

QTL name	Chromosome	Left marker	Right marker	Position (cM)	LOD score	Left CI	Right CI	PVE (%)	Pathotype
*qLr.iiwbr.6B.1*	6B	TaDArTAG006962	TaDArTAG009481	107	3.8493	105.84	108.19	14.219	Mixture of *P. triticina* pathotypes

### 3.6 Candidate gene identification

*In silico* analysis revealed a putative candidate gene for the seedling stage QTL exhibiting a higher PVE% i.e. *QLr.iiwbr.5BL.1* located at 525, 519, 604-525, 519754 Mb, encodes Annexin (TraesCS5B02G201700).

## 4 Discussion

Global research efforts have resulted in the identification, characterization, and mapping of more than 80 catalogued leaf rust resistance genes (Singh et al., 2024; Bariana et al., 2022). However numerous QTL and marker trait associations (MTAs) for resistance to leaf rust have also been reported (Kumar et al., 2022), but the rapid and continual evolution of the pathogen across diverse regions has rendered many of these genes ineffective, leaving only a limited number of *Lr* genes with sustained effectiveness. Therefore, dissecting these traits through QTL analysis and incorporating them into marker-based breeding programs will provide a valuable framework for developing effective strategies via marker-assisted selection (MAS) (Khan et al., 2022).

Seedling resistance, also referred to as all-stage resistance (ASR), is often characterized by a hypersensitive response and is typically governed by race-specific major genes (e.g., *Lr9*, *Lr26*). In the present study, the recombinant inbred line (RIL) population derived from a cross between W8627 and PBW343 exhibited a wide range of infection types (ITs) when challenged with P. *triticina* pathotypes 77-5, 77-1, and 12-7 at the seedling stage. Among these, pathotype 77-5 was the most virulent, inducing the highest IT scores across genotypes, followed by 77-1 and 12-7. The donor parent W8627 exhibited strong resistant to all three pathotypes (77-5, 77-1, and 12-7), confirming its broad- spectrum resistance reported previously. In contrast, PBW343 was susceptible; consistent with resistance breakdown in lines carrying *Lr26*. Segregation among the RILs suggested the presence of multiple resistance genes. Earlier studies, (Virdi et al., 2016) identified one recessive gene in W8627 conferring resistance to *P. triticina* (pathotype 77-5) and two complementary recessive genes against *Puccinia striiformis* f. sp. *tritici.* The present findings support this complex inheritance, highlighting W8627 as a valuable donor for resistance breeding (Sharma et al., 2025). However, the susceptibility of PBW343 underscores the vulnerability of all- stage resistance (ASR) to evolving races, reinforcing the need to combine seedling resistance with durable adult plant resistance (APR).

APR is generally considered more durable than ASR because it is typically conferred by race non-specific genes. Its durability lies in the fact that it cannot be easily overcome by single mutations in the pathogen’s genome, especially during its asexual reproduction cycle. The breakdown of PBW343’s resistance over time, once effective due to the *Lr26* gene, underscores the limitations of relying solely on major genes. This variety, despite its historical success in India’s NWPZ, now shows susceptibility to both leaf and stripe rust due to evolutionary pressures favoring virulent pathotypes (Sharma et al., 2021). These trends highlight the need for incorporating polygenic and non-race-specific resistance into breeding programs.

Field evaluations of the RIL population under artificial disease pressure revealed extensive variability among the RILs and the contrasting parents. Notably, some RILs showed complete immunity, suggesting transgressive segregation and the emergence of novel gene combinations surpassing parental resistance. These highly resistant lines are valuable for direct inclusion in breeding pipelines. Unlike ASR, APR does not usually provide complete immunity but slows disease progression. Known APR genes such as *Lr34*, *Lr46*, and *Lr67* have been linked to durable, non-race-specific resistance (Singh et al., 1998). By integrating field trials with seedling-stage screenings, the current study effectively identified lines likely to carry APR components, essential for achieving long-term disease control.

In this study, a high-density linkage map was developed, and QTL mapping revealed genomic regions associated with leaf rust resistance both at the seedling stage and adult plant resistance. Two QTLs associated with seedling resistance to leaf rust were identified across chromosomes 2B and 5B, in response to *P*. *triticina* pathotypes 77-1 and 77-5. A QTL *qLr.iiwbr.2B.1* was identified at 5 cM, with a LOD score of 8.08, explaining 13.26% of the phenotypic variation against the pathotype 77-1. This QTL was flanked by markers TaDArTAG006007 and TaDArTAG006082. Chromosome 2B, particularly its short arm (2BS), has been widely recognized as a hotspot for leaf rust resistance in wheat. The consistent identification of major leaf rust resistance loci on chromosome arm 2BS across diverse germplasm highlights its significance as a resistance gene rich region. In the present study, the QTL detected on 2BS aligns closely with previously reported genes and QTLs such as *Lr16*, *Lr23*, *Lr13*, *Lr35*, and *Lr48*, as well as slow-rusting QTLs like *QLr.usw-2BS*, *QLr.ksu-2BS*, and *QLr.hebau-2BS* (Ren et al., 2023). Notably, Our findings are consistent with recent reports that fine mapped *Lr16* using resistance gene analog (RGA)-based SNP markers, which demonstrated strong predictive power and utility for marker assisted selection (MAS). The co-localization of our QTL with multiple resistance loci suggests that this region may harbor tightly linked or clustered resistance genes contributing to both seedling and adult plant resistance. These findings reinforce the utility of chromosome 2BS as a target for pyramiding multiple resistance genes and support the use of diagnostic SNP markers to enhance the efficiency of MAS in wheat breeding programs aimed at durable leaf rust resistance. Collectively, these findings underscore the importance of the 2BS region as a target for breeding programs.

**Figure 6:**
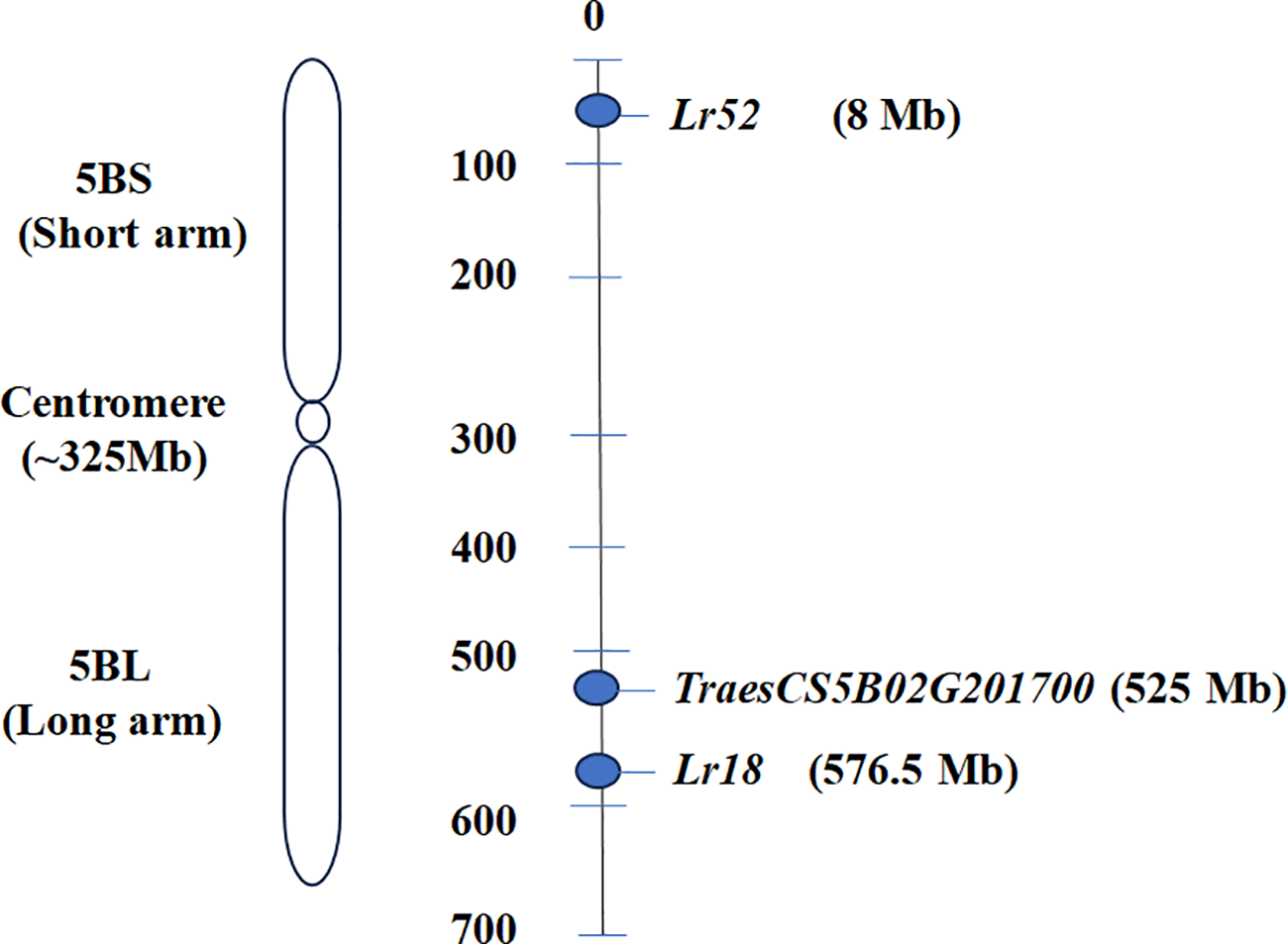
Physical position of leaf rust resistance loci on chromosome 5B.

A second QTL, *qLr.iiwbr.5B.1*, was detected in response to pathotype 77-5 on chromosome 5B, positioned at 32 cM, with a LOD score of 3.91 and PVE of 14.91%. The confidence interval for this QTL ranged from 31.5 cM to 32.5 cM, and it was flanked by the markers TaDArTAG007725 and TaDArTAG007717. Chromosome 5B is known to carry important resistance genes like *Lr52* (on 5BS) (Sadegahabad et al., 2017) and *Lr18* (on 5BL) (Hiebert et al., 2005), as well as QTLs like *QLr.cim-5BL* from resistant lines such as ‘Heller#1’ and ‘Dunkler’. These loci contribute to both seedling and adult plant resistance. The relative physical position of the leaf rust resistance genes are shown in the **Figure 6**. Interestingly, previous studies have also reported QTLs on the same chromosome, particularly for adult plant resistance (APR). For instance, Kumar et al. (2013) reported two minor but consistently expressed APR QTLs in the wheat variety Opata85 named *QLr.ccsu-5B.4* and *QLr.ccsu-5B.5* which showed stable resistance across multiple environments and years in India. Similarly, two APR QTLs were identified in the cultivar Jamestown, explaining up to 22.1% of phenotypic variation. Kolmer (2015b) also mapped a QTL on 5BL, designated as *QLr.cdl-5BL*, in a Thatcher³/Americano 25e population, which interacted additively with the known APR gene *Lr46*, providing enhanced resistance. Further, meta-QTL analysis by Soriano and Royo (2015) highlighted chromosome 5B as a hotspot for rust resistance by consolidating QTLs from multiple wheat genotypes including Americano 25e, Carberry, Capo, and Kariega into MQTL22 and MQTL23. The moderate PVE and tight confidence interval suggest that *qLr.iiwbr.5B.1* may be a stable locus with potential utility in breeding programs. Together, the QTLs identified in this study offer promising targets for marker-assisted selection (MAS) and the development of wheat cultivars with durable resistance to leaf rust.

The present study also identified a significant adult plant resistance (APR) QTL on Chromosome 6B, highlighting the polygenic and stage-specific nature of leaf rust resistance in wheat. A notable QTL, *qLr.iiwbr.6B.1*, was mapped on chromosome 6B at 107 cM, flanked by the markers TaDArTAG006962 and TaDArTAG009481. This QTL exhibited a LOD score of 3.84 and explained 14.21% of the phenotypic variation. The moderate effect size and non-race specific response suggest that *qLr.iiwbr.6B.1* may belong to the class of minor but durable APR genes. This finding aligns with earlier reports emphasizing the role of chromosome 6B in conferring long-lasting resistance to leaf rust. For instance, the QTL *QLr.caas-6BS.1* was mapped to the short arm of chromosome 6B in the Chinese wheat cultivar ‘Bainong 64’ (Ren et al., 2012), while *QLr.cimmyt-6BL.1* was identified on the long arm in the CIMMYT-derived variety ‘Pastor’ (Rosewarne et al., 2013). These observations reinforce the significance of both chromosome arms 6BS and 6BL in contributing to APR. In addition to QTLs, several known leaf rust resistance (*Lr*) genes are also located on chromosome 6B. The *Lr3* gene cluster, which includes alleles such as *Lr3a*, *Lr3bg*, and *Lr3ka*, is positioned on 6BL (Kolmer, 2015a). Furthermore, *Lr36*, a gene originally introgressed from the wild wheat relative *Aegilops speltoides*, is located on 6BS and confers resistance at the seedling stage (Ren et al., 2023). So, the QTL we found might be related to these known genes, or it could be a new source of resistance. This makes chromosome 6B a very useful region to focus on in breeding programs to combine (or pyramid) different types of resistance using molecular markers.

The non-overlapping location of *qLr.iiwbr.6B.1* relative to seedling-stage QTLs supports its independent inheritance, allowing for gene pyramiding to combine resistance from multiple stages without the risk of linkage drag. Furthermore, the observation of immune lines within the RIL population indicates the presence of transgressive segregants, likely arising from favorable recombination between seedling and adult resistance genes. From a breeding perspective, *qLr.iiwbr.6B.1* offers a valuable resource. The availability of flanking DArT markers enables marker-assisted selection (MAS) and facilitates its introgression into susceptible but agronomically elite cultivars like PBW343. Its incorporation could strengthen genetic defenses and contribute to durable, broad-spectrum resistance in wheat improvement programs.

Furthermore, we aim to refine the QTLs with significant phenotypic variance through computational analysis for the identification of putative candidate genes. For instance, *qLr.iiwbr.5B.1* encodes for annexin (TraesCS5B02G201700) which are known to play a positive role in mediating early defense signaling following pathogen attack in plants (Saad et al., 2020). Annexins are an evolutionarily conserved, multifunctional protein family characterized by annexin repeats with type II calcium-binding motifs, whose functional diversity arises from variation in their N-terminal regions (Xu et al., 2016). Rust infection triggers calcium biding and phospholipid interaction, leading to ROS accumulation, callose deposition, and salicylic acid biosynthesis; together these responses drive hypersensitive response and systemic acquired resistance, ultimately inhibiting rust hyphal growth and reducing disease severity as depicted in **Supplementary Figure 1**.Previous investigation has demonstrated the involvement of annexins in wheat stripe rust resistance (Shi et al., 2023). To our understanding, no prior studies have documented the involvement of annexins in leaf rust resistance in wheat. While the current evidence is based on *in silico* positioning and functional inference, further experimental validations are warranted to confirm annexins as bona fide candidate genes for leaf rust resistance and to explore their potential utility in breeding strategies aimed at enhancing broad-spectrum rust resistance in wheat.

## 5 Conclusion

In conclusion, this study revealed considerable variability for leaf rust resistance in RIL population, with some lines exhibiting complete immunity- surpassing even the resistant parent W8627- underscoring their potential for utilization in breeding. The new QTLs and candidate gene (Annexin) identified in this study warrant further validation across diverse genetic backgrounds and environments, coupled with functional characterization of the underlying genes will strengthen their utility in rust resistance breeding programs.

## Data Availability

The datasets presented in this study can be found in online repositories. The names of the repository/repositories and accession number(s) can be found in the article/**Supplementary Material**.

## Supplementary material

Supplementary Figure 1 Schematic representation of Annexin gene in rust resistance in wheat.
