# Supplementary figures and images for: QTL mapping for leaf rust resistance in bread wheat using PBW343/W8627 RIL population
Source: Front Plant Sci. 2026 Jul 1;17:1851529. doi: 10.3389/fpls.2026.1851529 (PMC13368715; doi:10.3389/fpls.2026.1851529)

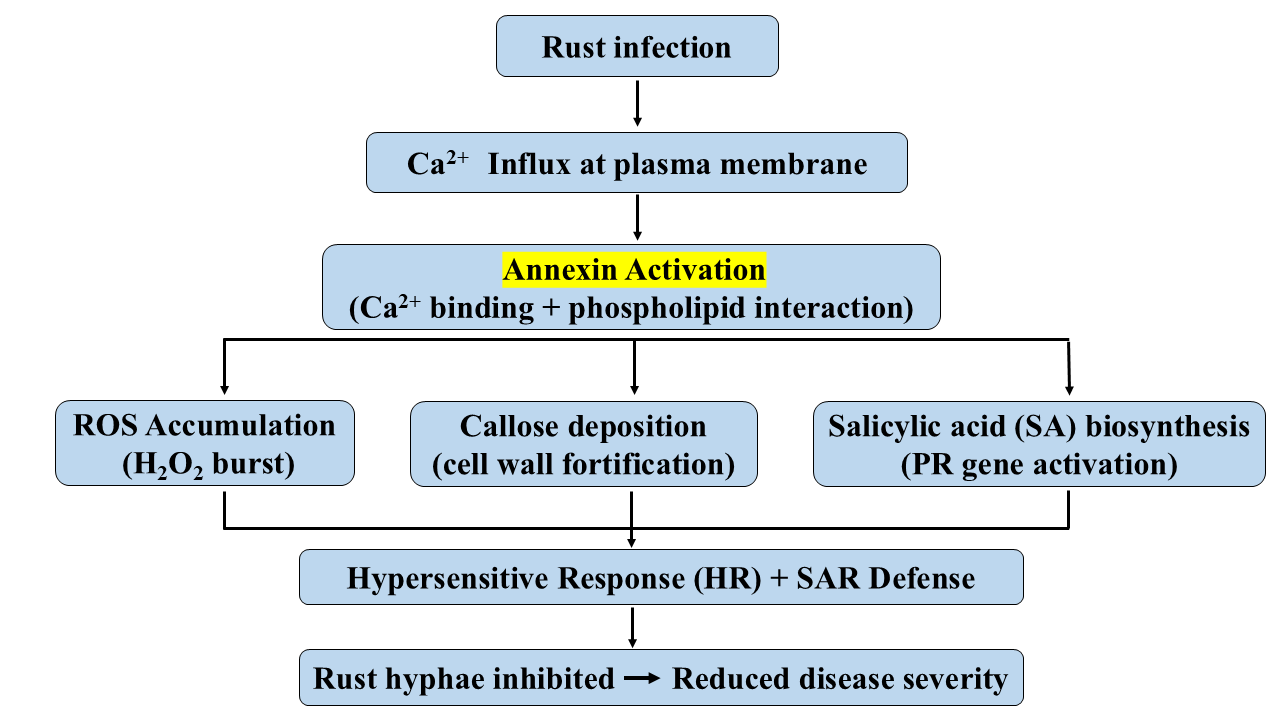


**Supplementary Figure S1:** Schematic representation of Annexin gene in rust resistance in wheat

Supplement: Supplementary Figure 1 — Schematic representation of Annexin gene in rust resistance in wheat. [file DataSheet1.doc]
